# Direct chemical vapor deposition of graphene on plasma-etched quartz glass combined with Pt nanoparticles as an independent transparent electrode for non-enzymatic sensing of hydrogen peroxide†

**DOI:** 10.1039/d0ra01963a

**Published:** 2020-05-28

**Authors:** Ning Li, Yawen Yuan, Jinglei Liu, Shifeng Hou

**Affiliations:** School of Chemistry and Chemical Engineering, Shandong University Jinan Shandong 250100 China; National Engineering and Technology Research Center for Colloidal Materials, Shandong University Jinan Shandong 250100 China shifenghou@sdu.edu.cn; Institute 53 of China North Industries Group Corporation Jinan Shandong 250031 China

## Abstract

In this work, chemical vapor deposition (CVD) method-grown graphene on plasma-etched quartz glass supported platinum nanoparticles (PtNPs/eQG) was constructed as an independent transparent electrode for non-enzymatic hydrogen peroxide (H_2_O_2_) detection. Graphene grown on quartz glass by the CVD method can effectively reduce the wrinkles and pollution caused by traditional transfer methods. The addition of the CF_4_ plasma-etched process accelerates the growth rate of graphene on quartz glass. The platinum nanoparticles (PtNPs) prepared by *in situ* sputtering have favorable dispersibility and maximize exposed active catalytic sites on graphene, providing performance advantages in the application of H_2_O_2_ detection. The resulting sensor's detection limit (3.3 nM, S/N = 3), detection linear range (10 nM to 80 μM) and response time (less than 2 s) were significantly superior to other graphene supported PtNPs materials in sensing of H_2_O_2_. In addition, the material preparation method was related to the non-transfer CVD method and *in situ* sputtering technology, allowing for the creation of independent electrodes without additional electrode modification processes. This primitive material preparation and electrode assembly process were promoted for the application and development of practical H_2_O_2_ sensors.

## Abstract 2

In this work, chemical vapor deposition (CVD) method-grown graphene on plasma-etched quartz glass supported platinum nanoparticles (PtNPs/eQG) was constructed as an independent transparent electrode for non-enzymatic hydrogen peroxide (H_2_O_2_) detection.

## Graphical abstract

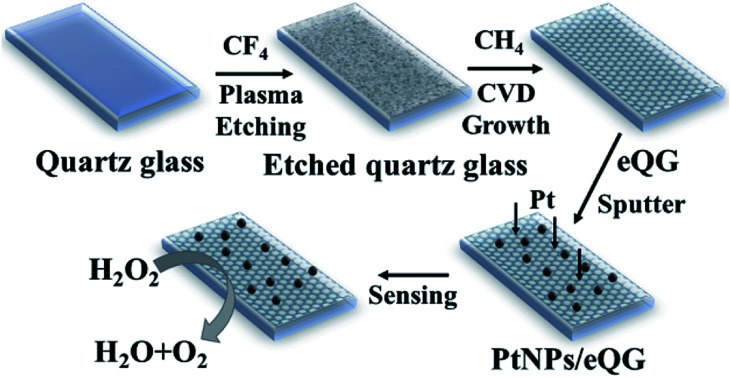

## 1. Introduction

Hydrogen peroxide (H_2_O_2_) is an important intermediate in the chemical industry and a significant indicator of major diseases such as cancer, Alzheimer's disease and Parkinson's disease.^1,2^ The accurate detection of low concentrations of H_2_O_2_ has important practical significance in the fields of clinical medicine, environment and food testing. In recent years, many methods have been developed for the effective detection of H_2_O_2_, including the chemiluminescence method,^3^ spectral method,^4^ chromatographic method,^5^ electrochemical method^6^ and so on. Of these, non-enzymatic electrochemical H_2_O_2_ sensors were especially widely used because of their simple equipment, easy miniaturization, high sensitivity, good selectivity and fast response.^7–9^

The sensitivity, selectivity and stability of electrochemical sensors are strongly dependent on the structure and performance of the electrode materials.^10^ Graphene, a two-dimensional carbon nanomaterial,^11^ is widely used as a transparent electrode because of its large surface area, excellent conductivity, ultra-high optical transparency and chemical stability.^12,13^ At the same time, noble metal nanoparticles, with large surface-to-volume ratio, extraordinary conductivity and high catalytic performance, have been widely applied in the electrochemical sensing fields.^14–19^ What's more, platinum nanoparticles (PtNPs) have higher catalytic ability and stability for the detection of H_2_O_2_ due to their lower oxidation/reduction overvoltage.^20^ Therefore, graphene supported PtNPs has attracted great attention for non-enzymatic detection of H_2_O_2_ due to favorable properties of graphene as transparent electrodes and ultra-high detection performance of PtNPs.^21^ For example, Selvakumar Palanisamy^22^*et al.* reported a single step electrochemical fabrication of a platinum nanoparticle decorated reduced graphene oxide (RGO–PtNPs) composite for enhanced electrochemical sensing of H_2_O_2_. It was linear over the concentration ranging from 0.05 to 750.6 mM with the limit of detection of 16 nM. Their study conclusively reveals the feasibility of the application of graphene nano-platinum composites in electrochemical H_2_O_2_ sensing.

Nevertheless, it is still a tremendous challenge to prepare high-quality, large-scale and non-polluting graphene for electrochemical sensing.^23^ For example, graphene prepared by mechanical exfoliation^24^ and redox methods^25^ are agglomerated and have functional group residue, which leads to a decrease in the conductivity and sensing properties of sensors.^26,27^ At present, graphene grown by chemical vapor deposition (CVD) method has become the most promising material due to their large specific surface area and high crystalline quality.^28,29^ However, this additional transfer process is extremely prone to resulting in wrinkles, cracks, contamination and defects in graphene. This greatly limits CVD graphene from developing the proper electrochemical properties.^30,31^

Direct CVD growth of graphene on the target substrate has immensely aroused research enthusiasm.^32^ The direct growth of graphene on the target substrate can reduce defects and contamination caused by complex transfer processes and better preserve the physical and chemical properties of graphene. The excellent electrochemical properties of graphene grown on target substrate can improve its potential application in the field of electrochemistry. High-quality graphene produced by non-transfer CVD method can be directly used in self-assembly electrode devices, which is convenient in electrochemistry and promoting the industrialization of CVD graphene.^33^

In this paper, graphene was directly grown on the surface of quartz glass by atmospheric pressure chemical vapor deposition (APCVD). The effect of plasma etching of quartz glass on the growth behavior of graphene was studied.^34^ Graphene grown on plasma-etched quartz glass (eQG) combined with sputtered Pt nanoparticles (PtNPs) were used as independent electrodes to assemble electrochemical sensors for non-enzymatic detection of low concentration of H_2_O_2_.

## 2. Experimental section

### 2.1 Chemicals

Quartz glass (SiO_2_) of size of 10 × 5 × 0.5 mm was purchased from Hefei Kejing Crystal Technology CO., LTD (Hefei, China). Hydrogen (H_2_), methane (CH_4_), carbon tetrafluoride (CF_4_), nitrogen (N_2_) and argon (Ar) gases with 99.99% purity were all purchased from Jinan Deyang Special Gas Company (Jinan, China). Hydrogen peroxide (H_2_O_2_) with a 30% mass fraction, acetone (CH_3_COCH_3_), sulfuric acid (H_2_SO_4_, with a 98% mass fraction), sodium dihydrogen phosphate dihydrate (NaH_2_PO_4_·2H_2_O), disodium hydrogen phosphate (Na_2_HPO_4_), ascorbic acid (AA), dopamine (DA) and uric acid (UA) were purchased from Sinopharm Chemical Regent Company (Shanghai, China). Phosphate buffer solution (0.1 mol L^−1^, pH = 7.2) was a mixture of NaH_2_PO_4_ solution (0.1 mol L^−1^) and Na_2_HPO_4_ solution (0.1 mol L^−1^). All reagents used in the experiments were of analytical grade and used without further purification process. Deionized water with a resistivity of 18.25 MΩ cm was used in all aqueous solutions.

### 2.2 Apparatus

The compact etcher (RIE-1C) was obtained from SAMCO Inc. (Kyoto, Japan). The atmospheric pressure chemical vapor deposition (APCVD) equipment was purchased from Xicheng company (Xiamen, China). Vacuum sputter equipment (DESK V Cold Sputter) was purchased from Denton Vacuum Company (Moorestown, USA). The platinum target with 99.999% purity was obtained from GRIKIN Advanced Material Company (Beijing, China). The electrochemical workstation (CHI-660C), containing a standard three-electrodes system, was purchased from Shanghai ChenHua Instrument Company (Shanghai, China).

The morphologies and structures of the samples were characterized by scanning electron microscopy (SEM-SU8010, Hitachi, Japan), Raman spectrometer (PHS-3C, Horbin, France), UV-Visible Spectrophotometer (TU-1901, PERSEE, Beijing, China). X-ray photoelectron spectrometer (XPS, ThermoFisher K-Alpha, USA) and high resolution transmission electron microscope (HRTEM, JEM-2100, Japan).

### 2.3 Preparation of PtNPs/eQG

#### Graphene grown on plasma-etched quartz glass (eQG)

Graphene was grown on quartz glass substrate using the atmospheric pressure chemical vapor deposition (APCVD) method. Firstly, the quartz glass was ultrasonically washed with ethanol, acetone, and ultrapure water for 30 minutes in that order. After drying, the quartz glass was immersed in a piranha solution (*V*_H_2_SO_4__/*V*_H_2_O_2__ = 7 : 3) and heated to 120 °C for 30 min. It was then rinsed with ultrapure water and dried with nitrogen. The cleaned quartz glass was then placed in the compact etcher to etch with 30 sccm CF_4_ for a designated period of time. Then the plasma-etched quartz glass was placed in silica tube under a mixed atmosphere of argon (250 standard-state cubic centimeter per minute, sccm) and hydrogen (40 sccm) and heated to 1030 °C. A certain amount of CH_4_ gas (11 sccm) was subsequently introduced into the reactor at 1030 °C for a certain period of time. Then, the silica tube was cooled to room temperature within 30 minutes. The sample was recorded as graphene grown on plasma-etched quartz glass (eQG). Graphene was grown on the surface of quartz glass for 180 min after etching for 5 min, 10 min and 15 min to confirm the effect of etching time on the growth of graphene. The effect of growth time of graphene was also studied: the growth time of graphene on quartz glass plasma-etched for 10 min was 150, 1180 and 210 min, as denoted by eQG_150_, eQG_180_ and eQG_210_, respectively.

#### Pt nanoparticles/graphene grown on plasma-etched quartz glass (PtNPs/eQG)

Graphene grown on plasma-etched quartz glass (eQG) finally, eQG was moved into the vacuum sputter equipment with the plasma-etched side of the quartz glass facing up. The power of the sputter equipment was 50 W and the sputtering time was 5 min. The prepared Pt nanoparticles/graphene grown on plasma-etched quartz glass is denoted as PtNPs/eQG. The samples of sputtering Pt nanoparticles on graphene grown on quartz glass at growth times of 150, 180 and 210 min were denoted PtNPs/eQG_150_, PtNPs/eQG_180_ and PtNPs/eQG_210_, respectively.

### 2.4 Preparation of contrast electrodes

#### Graphene grown on quartz glass (QG)

For comparison, graphene grown on quartz glass (QG) was prepared by immediately placing cleaned quartz glass in silica tubes without etching under the same conditions for graphene growth.

#### Platinum nanoparticles loaded on plasma-etched quartz glass (PtNPs/eQ)

As mentioned above, the cleaned quartz glass was placed in the compact etcher to etch with 30 sccm CF_4_ for 10 min labeled as platinum nanoparticles/plasma-etched quartz glass (PtNPs/eQ) were created by directly sputtering PtNPs on plasma-etched quartz glass for 5 min.

### 2.5 Electrochemical test

**Scheme 1:**
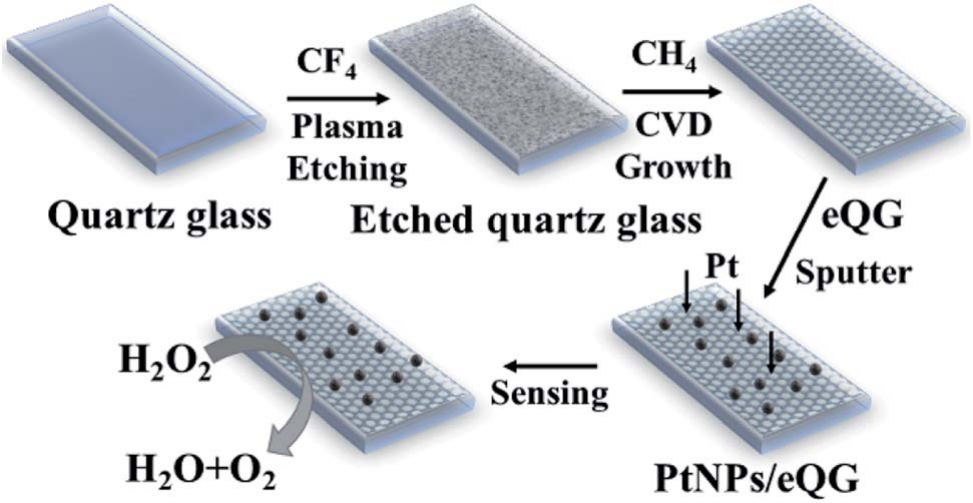
Schematic of PtNPs/eQG synthesis.

Firstly, 20 mL phosphate buffer solution (0.1 mol L^−1^, pH = 7.2) was added to a self-made electrochemical test system that includes a 25 mL beaker and a cover plate. The three-electrode electrochemical system contained a saturated calomel electrode as the reference electrode, PtNPs/eQG as the working electrode and platinum plate as the counter electrode. Then nitrogen was injected for 30 min to discharge oxygen. Cyclic voltammogram (CV) measurements were implemented from −0.3 V to 0.8 V both in the presence and the absence of 10 μM H_2_O_2_ with a scan rate of 50 mV s^−1^. The amperometric *i*–*t* curve was measured with 25 μL different concentrations of H_2_O_2_ at regular intervals of 50 s. Ascorbic acid (AA), dopamine (DA) and uric acid (UA) were intermittently added to detect the anti-interference performance (Scheme 1).

## 3. Results and discussion

### 3.1 Characterization of PtNPs/eQG

**Fig. 1:**
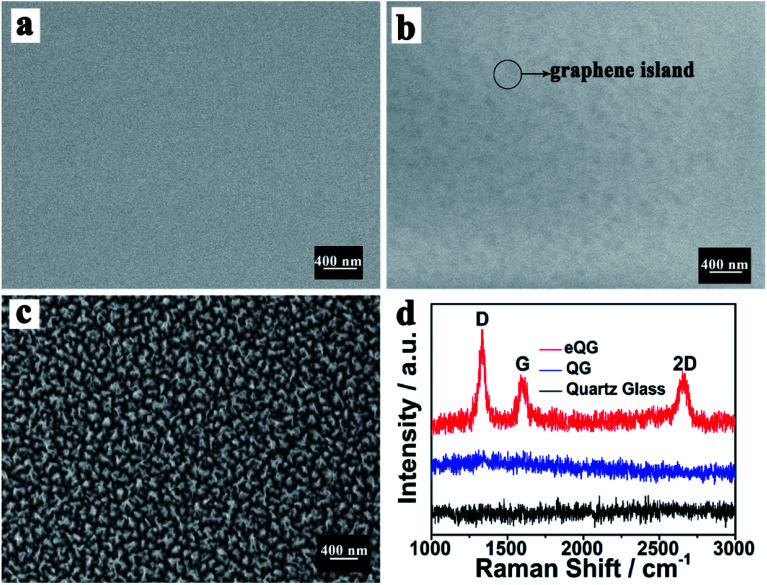
SEM images of (a) quartz glass, (b) QG and (c) eQG and (d) Raman spectrum of quartz glass, QG and eQG.

The morphology of quartz glass, QG and eQG was determined by SEM. These are shown in Fig. 1a–c. After graphene growth for 180 min, the glass surface was covered with graphene islands but was not substantially covered, as shown in Fig. 1b. After CF_4_ plasma etching, the increased roughness of quartz glass added active sites to improve carbon atom deposition and domain nucleation, as shown in Fig. 1c. The CF_4_ plasma-etched quartz glass surface served as the active surface for graphene growth.

The growth mechanism of graphene film on plasma-etched quartz glass is similar to that on heat-treated quartz glass.^35^ As shown in Fig. 1d, the Raman spectrum of graphene grown on eQG showed D (1323 cm^−1^), G (1600 cm^−1^) and 2D (2650 cm^−1^) peaks of graphene.^36–38^ The ratio of G peak to 2D peak was about 1 and the peak width of the 2D peak was greater than 50 cm^−1^, which shows that there were a few layers of graphene. The D peak at 1323 cm^−1^ shows the defect of graphene, which indicates that the defect structure of graphene was easier to form when quartz glass was treated by etching. In contrast, there was no obvious characteristic peak of graphene on the non-plasma-etched quartz glass substrate for the same growth time.

The effect of the etching process on the growth of graphene on quartz glass was verified by testing different etching times. From Fig. S1,† with the prolongation of etching time, the nanoscale gullies created by plasma-etching became larger and larger, which showed the increase of roughness. With increased etching time, the characteristic peak of graphene (Fig. S1e†) still appeared in the Raman spectrum with the ratio of peak intensity of G and 2D being about 1. This shows that the rough surface was conducive to the growth of a few layers of graphene and has good crystal quality. However, as the etching time continued to increase, *I*_G_/*I*_2D_ became larger (Fig. S1f†), indicating an increase in the number of graphene layers. It is speculated that the large roughness of the quartz substrate will increase the number of carbon atoms deposited and lead to the accumulation of graphene. Due to the ideal properties of a few layers of graphene, we chose quartz glass etching for 10 min as the substrate for graphene growth.

The growth mechanism of graphene film on plasma-etched quartz glass was similar to that on heat-treated quartz glass.^36^ The plasma-etching of the quartz substrate could increase the roughness of the quartz glass surface as shown in Fig. 1c. The defects provided by the roughness would provide more opportunities for graphene domain nucleation. A plurality of adjacent graphene islands was joined together to form a continuous graphene film.

After the 180 min CVD growth process, the untreated quartz glass did not join the graphene islands into graphene films and did not show significant peak positions in the Raman spectrum measurements. The quartz glass after being plasma-etched had obvious Raman spectral peak positions, indicating that the continuous graphene film can grow quickly and effectively on the surface of plasma-etched quartz glass.

**Fig. 2:**
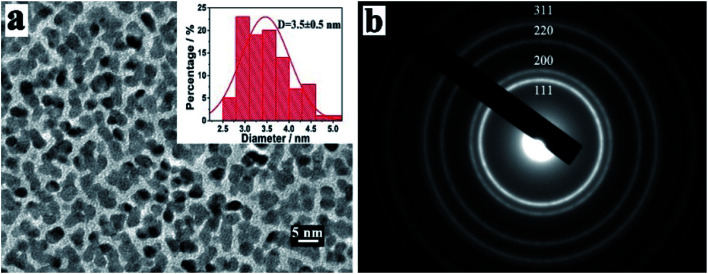
(a) HRTEM images and (b) corresponding SAED pattern of PtNPs.

Furthermore, the morphology and structure of platinum nanoparticles (PtNPs) prepared by vacuum sputter equipment were characterized by HRTEM and corresponding selected area electron diffraction (SAED) pattern, as depicted in Fig. 2. As shown in the HRTEM image of Fig. 2a, the sputtered PtNPs had good dispersion with the average size being about 3.5 ± 0.5 nm (the histogram of size distribution was inset in Fig. 2a). The SAED patterns of PtNPs showed multiple diffraction rings, which were attributed to the reflection lattice plane of Pt (111), (200), (220) and (311), as shown in Fig. 2b. The results confirmed the successful preparation of PtNPs with good dispersion and small sizes by sputtering technology.

The surface composition and element characterization of PtNPs/eQG were analyzed by X-ray photoelectron spectroscopy (XPS), as depicted in Fig. S2.† As shown in Fig. S2a,† there were four elements in the XPS spectra of PtNPs/eQG: platinum, carbon, oxygen and silicon. The spectra of XPS existed the characteristic peaks of Si 2p (104.5 eV) and Si 2s (150 eV), which was indicative of the existence of the SiO_2_ phase in PtNPs/eQG. In addition, the presence of SiO_2_ could be further confirmed by the O 1s XPS peak at 532.8 eV, which was regarded as the oxygen species in the SiO_2_. As shown in Fig. S2b,† the highest peak of C 1s in the sample was 284.6 eV, corresponding to the C=C bond. The remarkable carbon peak of sp^2^ hybrids indicates that the sample had obvious graphite structure. The C 1s peak in the sample could be divided into four peaks, with the other three peaks at 285.8 eV, 287.6 eV and 289.1 eV representing C–C, C–O and O=C–O bonds, respectively. Fig. S2c† showed the splitting of Pt 4f of PtNPs/eQG to two peaks at 70.9 eV and 74.3 eV, corresponding to Pt (0) 4f_7/2_ and Pt (0) 4f_5/2_, respectively,^39^ indicating the feasibility of nanoparticle sputtering technology in applications.

The effects of different growth time were investigated by comparing the Raman and UV-Vis transmission spectra of graphene grown on quartz glass with etching time of 10 min and growth time of 150 min, 180 min and 210 min. As shown in Fig. S3a,† the sharp G peak indicates that the graphene prepared under these three conditions had good crystal quality. The sharp D peak indicates that the etching of quartz glass accelerated the formation of the defect structure of graphene. With the increase in growth time, the number of graphene layers increased. This is perhaps due to the aggregation and agglomeration of graphene that follows from increases in the amount of deposited carbon atoms. As shown in Fig. S3b,† when the other growth conditions were the same, the growth time of 150 min had a high transmittance of 93.7%. By comparing the transmittance trends of different growth times, it could be deduced that the longer the growth time, the lower the transmittance. By extending the growth time to 210 min, the transmittance was only at about 89.0%. After sputtering Pt nanoparticles, the transmissivity of PtNPs/eQG_180_ in Fig. S3b† was reduced to 86.4%. It could be seen from the inseted figure that the electrode had excellent transmittance, making the electrode ideal for use in sensing, optoelectronic devices and other fields.

### 3.2 Electrochemical behavior of PtNPs/eQG

Fig. S4† shows the electrochemical cyclic voltammetric (CV) curve of Pt nanoparticles/graphene grown on plasma-etched quartz glass for different growth times. With the addition of H_2_O_2_, PtNPs/eQG_150_ and PtNPs/eQG_210_ increased current value without any peaks in this voltage window. The CV curve of PtNPs/eQG_180_ shows significant peaks at −0.05 V and 0.60 V in the presence of H_2_O_2_ (red solid line). This indicates the best electrochemical response electrode for H_2_O_2_ detection is PtNPs/eQG_180_, meaning that the optimal preparation time for graphene growth is 180 min. Therefore, the transparent electrode prepared under this condition was used as the working electrode for subsequent electrochemical performance measurements. We'll use PtNPs/eQG to denote PtNPs/eQG_180_.

**Fig. 3:**
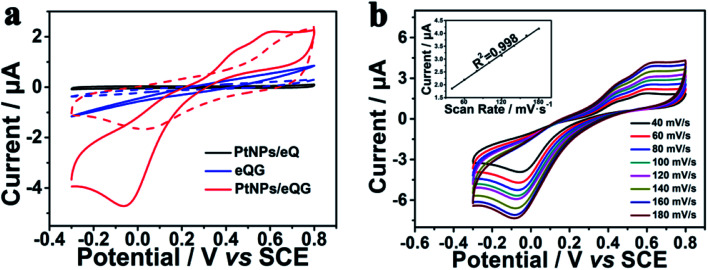
(a) Cyclic voltammogram curves of PtNPs/eQ (black line), eQG (blue line) and PtNPs/eQG (red line) in the presence (solid line) and the absence (dashed line) of 10 μM H_2_O_2_ with a scan rate of 50 mV s^−1^. (b) Cyclic voltammogram curves of PtNPs/eQG in phosphate buffer solution containing 10 μM H_2_O_2_ at various scan rates with regular intervals of 20 mV s^−1^, inset (b) was the fitting curve of current value and sweep rate at 0.60 V.

The electrochemical behavior of Pt nanoparticles/graphene grown on plasma-etched quartz glass (PtNPs/eQG) towards the sensing of H_2_O_2_ was explored using CV tests. Fig. 3a exhibits CV curves of platinum nanoparticles loaded on plasma-etched quartz glass (PtNPs/eQ, black line), graphene grown on plasma-etched quartz glass (eQG, blue line) and platinum nanoparticles/graphene grown on plasma-etched quartz glass (PtNPs/eQG, red line) in the presence (solid line) and absence (dashed line) of 10 μM H_2_O_2_. The voltammogram ranged from −0.3 V to 0.8 V at a scan rate of 50 mV s^−1^. As shown, PtNPs/eQ and eQG had increased current value with the addition of H_2_O_2_. The CV curve of PtNPs/eQG shows higher capacitance and significant peaks of −0.05 V and 0.60 V in the presence of H_2_O_2_ (red solid line), which indicates PtNPs/eQG had an exceptional electrochemical response to low concentration of H_2_O_2_. In summary, comparing the response CV curves of different materials to H_2_O_2_, PtNPs/eQG had an impeccable electrochemical response to low concentration of H_2_O_2_.

In order to demonstrate the charge transport characteristics of PtNPs/eQG in the process of electrochemical sensing H_2_O_2_, cyclic voltammetric curves with various scanning rates (40 mV s^−1^–180 mV s^−1^) were recorded in Fig. 3b. When the scanning rate increased, the redox peak current changed significantly. Inset Fig. 3b shows the linear relationship between the peak current of PtNPs/eQG at 0.60 V and the scanning rate. The linear regression equation is *I* = 0.0168 × *ν* + 1.1829 (μA, mV s^−1^, correlation coefficient *R*^2^ = 0.998), indicating the dynamics control was an adsorption process.

### 3.3 Amperometric determination of H_2_O_2_

**Fig. 4:**
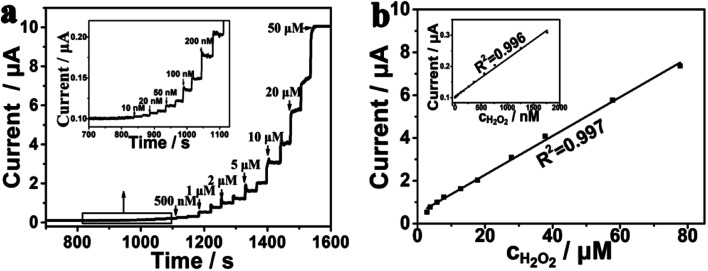
(a) Amperometric *i*–*t* curve for PtNPs/eQG with the injection of H_2_O_2_ in N_2_-saturated phosphate buffer solution (0.1 mol L^−1^, pH = 7.2) at 0.60 V with regular intervals of 50 s (b) and its internal illustration are plots of current valve *versus* low and high concentrations of H_2_O_2_.

Amperometric *i*–*t* curve was used to test the electrochemical performance of the PtNPs/eQG toward H_2_O_2_ by adding different concentrations of H_2_O_2_ solution into the stirring system at 0.60 V. The interval was 50 seconds and H_2_O_2_ was injected twice. As shown in Fig. 4a, the response current continuously jumped and rapidly increased to reach a steady state value within 2 s when the concentration of H_2_O_2_ increased from 10 nM to 50 μM. The results show PtNPs/eQG had excellent electrochemical performances for H_2_O_2_ detection.

The response current varied with the concentration of H_2_O_2_ as shown in Fig. 4b. In the range of 10–2000 nM, the response current had a good linear relationship with the H_2_O_2_ concentration, which is in accordance of the formula *I* = 0.10392 + 0.00012 × *c* (μA, nM, *R*^2^ = 0.996). In the range of 2–80 μM, the response current value had a strong linear relationship with the H_2_O_2_ concentration, in accordance of the formula *I* = 0.46593 + 0.09077 × *c* (μA, μM, *R*^2^ = 0.998). In the case of a signal-to-noise ratio of 3, the detection limit was 3.3 nM, which shows the good sensitivity of PtNPs/eQG for the detection of ultralow concentration of H_2_O_2_.

The comparison of detection limits and linear ranges between graphene-supported PtNPs towards H_2_O_2_ detection were shown in Table 1. PtNPs/eQG as an independent electrode had lower detection limit and faster phase response time than other graphene-supported PtNPs modified electrode. We believed that the excellent electrocatalytic activity of PtNPs/eQG nanocomposites can be attributed to the active role of CVD graphene. First, the larger specific surface area of CVD graphene provides more active sites by increasing PtNPs loading, promote the dispersion and expand the active region. Second, the excellent conductivity of graphene and the synergistic effect between Pt atoms and graphene provides for the rapid transfer of electrons from the reaction site to the electrode. Third, as the framework of PtNPs, graphene can protect the nano structure from agglomeration, deformation and collapse in harsh electrochemical test environments. Therefore, PtNPs/eQG is a preeminent independent electrode material for preparing H_2_O_2_ sensors with low detection limit, short response time and wide linear range.

**Table 1:** Comparison of the performance of graphene-supported PtNPs for H_2_O_2_ determination

Electrode materials	Detection limit (μM)	Linear range (μM)	Reference
Pt/GN^a^/GCE	0.8	2.5–6650	40
GN-Pt^b^/GCE	0.5	2–710	21
Pt-IL-pGR/GCE	0.42	10–4000	41
PdPt NCs@SGN/GCE	0.3	1–300	42
RGO-PT-Pt	0.26	1–100	43
PtNPs-CDs/IL-GO/GCE	0.1	1–900	44
RGO–PtNPs/GCE	0.016	0.05–875	22
PtNPs/eQG	0.0033	0.01–80	This work

a Microwave-assisted synthesis of Pt/graphene nanocomposites.

b Graphene–Pt nanocomposite.

The selectivity of PtNPs/eQG for H_2_O_2_ detection was investigated in Fig. S5.† The amperometric response of H_2_O_2_ (0.05 mmol L^−1^) and dopamine (DA, 0.5 mmol L^−1^), ascorbic acid (AA, 0.5 mmol L^−1^) and uric acid (UA, 0.5 mmol L^−1^) at 0.60 V was measured. It can be seen from Fig. S5† that the response current caused by DA, AA and UA was negligible compared to the response current caused by H_2_O_2_. The results show that PtNPs/eQG had strong anti-interference performances. This makes it an ideal candidate to be applied to the low-concentration qualitative and quantitative detection of H_2_O_2_ in the determination of actual biological samples.

## 4. Conclusions

In conclusion, we have successfully prepared CVD-grown graphene on plasma etched quartz glass (eQG) and loaded Pt nanoparticles (PtNPs) to prepare composite materials, and used it as an independent transparent electrode for non-enzymatic detection to H_2_O_2_. The growth rate of graphene on quartz glass was accelerated by adding a CF_4_ plasma etched process. The electrochemical properties of CVD graphene without transfer and the active sites exposed of PtNPs contributed to the catalytic performance of PtNPs/eQG for H_2_O_2_ detection. The sensor has an ultra-low detection limit of 3.3 nM (S/N = 3), lower than previous similar sensors. The combination of CVD-grown graphene without transfer and *in situ* sputtering technology is beneficial in the practical application of non-enzymatic detection of H_2_O_2_.

## Conflicts of interest

There are no conflicts to declare.

## Supplementary Material

RA-010-D0RA01963A-s001

## References

[cit1] Jia N., Huang B., Chen L., Tan L., Yao S. (2014). Sens. Actuators, B.

[cit2] Masuoka N., Wakimoto M., Ubuka T., Nakano T. (1996). Clin. Chim. Acta.

[cit3] Hu Y., Zhang Z., Yang C. (2007). Anal. Chim. Acta.

[cit4] Chai X. S., Hou Q. X., Luo Q., Zhu J. Y. (2004). Anal. Chim. Acta.

[cit5] Di Furia F., Prato M., Quintily U., Salvagno S., Scorrano G. (1984). Analyst.

[cit6] Ni Y., Song H., Kokot S. (2013). Electroanalysis.

[cit7] Priya C., Sivasankari G., Sriman Narayanan S. (2012). Colloids Surf., B.

[cit8] Liu M., Liu R., Chen W. (2013). Biosens. Bioelectron..

[cit9] Song M.-J., Hwang S. W., Whang D. (2010). Talanta.

[cit10] Qureshi A., Kang W. P., Davidson J. L., Gurbuz Y. (2009). Diamond Relat. Mater..

[cit11] Geim A. K., Novoselov K. S. (2007). Nat. Mater..

[cit12] Castro Neto A. H., Guinea F., Peres N. M. R., Novoselov K. S., Geim A. K. (2009). Rev. Mod. Phys..

[cit13] Zhang X., Bao Y., Bai Y., Chen Z., Li J., Feng F. (2019). Electrochim. Acta.

[cit14] Wang J. (2012). Microchim. Acta.

[cit15] Guler M., Turkoglu V., Bulut A., Zahmakiran M. (2018). Electrochim. Acta.

[cit16] Yuan Y., Zheng Y., Liu J., Wang H., Hou S. (2017). Microchim. Acta.

[cit17] Zhang C., Li C., Yu J., Jiang S., Xu S., Yang C., Liu Y. J., Gao X., Liu A., Man B. (2018). Sens. Actuators, B.

[cit18] Zhang C., Li Z., Jiang S. Z., Li C. H., Xu S. C., Yu J., Li Z., Wang M. H., Liu A. H., Man B. Y. (2017). Sens. Actuators, B.

[cit19] Li Z., Jiang S., Huo Y., Ning T., Liu A., Zhang C., He Y., Wang M., Li C., Man B. (2018). Nanoscale.

[cit20] Shahdost-fard F., Salimi A., Khezrian S. (2014). Biosens. Bioelectron..

[cit21] Xu F., Sun Y., Zhang Y., Shi Y., Wen Z., Li Z. (2011). Electrochem. Commun..

[cit22] Palanisamy S., Lee H. F., Chen S.-M., Thirumalraj B. (2015). RSC Adv..

[cit23] Pumera M., Ambrosi A., Bonanni A., Chng E. L. K., Poh H. L. (2010). Trac. Trends Anal. Chem..

[cit24] Chen J., Duan M., Chen G. (2012). J. Mater. Chem..

[cit25] Pei S., Cheng H.-M. (2012). Carbon.

[cit26] Yi M., Shen Z. (2015). J. Mater. Chem. A.

[cit27] Rozada R., Paredes J. I., Villar-Rodil S., Martínez-Alonso A., Tascón J. M. D. (2013). Nano Res..

[cit28] Dao V.-D., Nang L. V., Kim E.-T., Lee J.-K., Choi H.-S. (2013). ChemSusChem.

[cit29] Yuan Y., Zhang F., Wang H., Liu J., Zheng Y., Hou S. (2017). RSC Adv..

[cit30] Gomez De Arco L., Zhang Y., Schlenker C. W., Ryu K., Thompson M. E., Zhou C. (2010). ACS Nano.

[cit31] Liang X., Sperling B. A., Calizo I., Cheng G., Hacker C. A., Zhang Q., Obeng Y., Yan K., Peng H., Li Q., Zhu X., Yuan H., Hight Walker A. R., Liu Z., Peng L.-m., Richter C. A. (2011). ACS Nano.

[cit32] Xu S., Man B., Jiang S., Yue W., Yang C., Liu M., Chen C., Zhang C. (2014). Nanotechnology.

[cit33] Chen Z., Qi Y., Chen X., Zhang Y., Liu Z. (2019). Adv. Mater..

[cit34] Yang H., Huang L., Chang Q. H., Ma Z. J., Xu S. H., Chen Q., Shi W. Z. (2014). J. Phys. D: Appl. Phys..

[cit35] Medina H., Lin Y.-C., Jin C., Lu C.-C., Yeh C.-H., Huang K.-P., Suenaga K., Robertson J., Chiu P.-W. (2012). Adv. Funct. Mater..

[cit36] Chen J., Wen Y., Guo Y., Wu B., Huang L., Xue Y., Geng D., Wang D., Yu G., Liu Y. (2011). J. Am. Chem. Soc..

[cit37] Sun J., Chen Z., Yuan L., Chen Y., Ning J., Liu S., Ma D., Song X., Priydarshi M. K., Bachmatiuk A., Rümmeli M. H., Ma T., Zhi L., Huang L., Zhang Y., Liu Z. (2016). ACS Nano.

[cit38] Chen Z., Guo X., Zhu L., Li L., Liu Y., Zhao L., Zhang W., Chen J., Zhang Y., Zhao Y. (2018). J. Mater. Sci. Technol..

[cit39] Mayavan S., Sim J.-B., Choi S.-M. (2012). J. Mater. Chem..

[cit40] Zhang F., Wang Z., Zhang Y., Zheng Z., Wang C., Du Y., Ye W. (2012). Int. J. Electrochem. Sci..

[cit41] Zhang H., Bo X., Guo L. (2016). Electrochim. Acta.

[cit42] Fu Y., Huang D., Li C., Zou L., Ye B. (2018). Anal. Chim. Acta.

[cit43] Huang Y., Xue Y., Zeng J., Li S., Wang Z., Dong C., Li G., Liang J., Zhou Z. (2018). Mater. Sci. Eng. C.

[cit44] Chen D., Zhuang X., Zhai J., Zheng Y., Lu H., Chen L. (2018). Sens. Actuators, B.

