# Supplementary material for: Direct chemical vapor deposition of graphene on plasma-etched quartz glass combined with Pt nanoparticles as an independent transparent electrode for non-enzymatic sensing of hydrogen peroxide
Source: RSC Adv. 2020 May 28;10(35):20438–44. doi: 10.1039/d0ra01963a (PMC9054246; doi:10.1039/d0ra01963a)
Supplement: RA-010-D0RA01963A-s001 [file RA-010-D0RA01963A-s001.pdf]

Electronic Supplementary Material (ESI) for RSC Advances.
This journal is © The Royal Society of Chemistry 2020

Electronic Supplementary Material for

Direct Chemical Vapor Deposition Graphene on Plasma-etched Quartz Glass Combined with Pt Nanoparticles as an Independent Transparent Electrode for Non-enzymatic Sensing of Hydrogen Peroxide

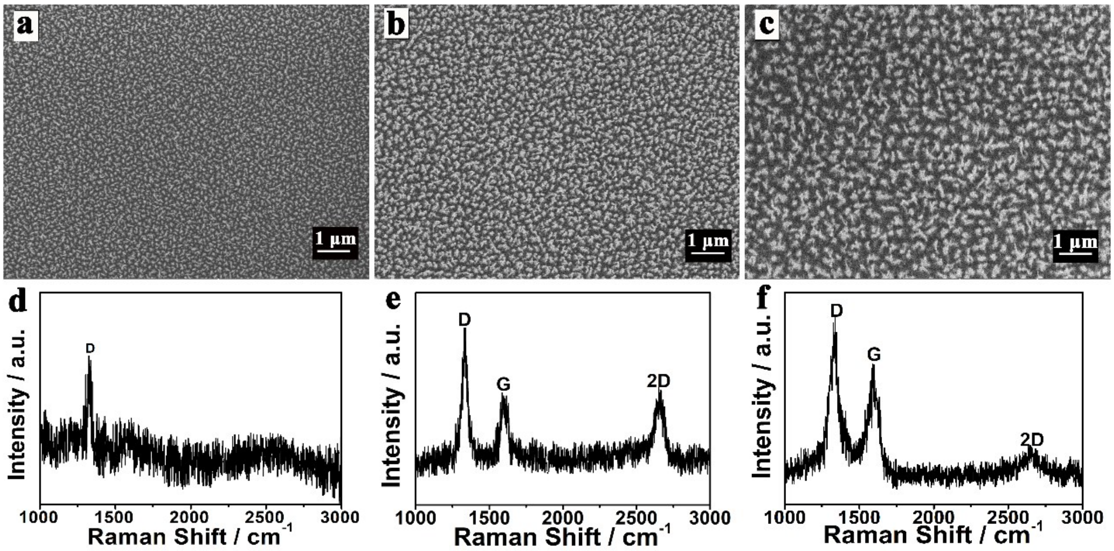

Fig. S1 (a, b, c) SEM images and (d, e, f) Raman spectrum of graphene grown on etched quartz glass (eQG) for etching times of 5 min (a, d), 10 min (b, e) and 15 min (c, f).

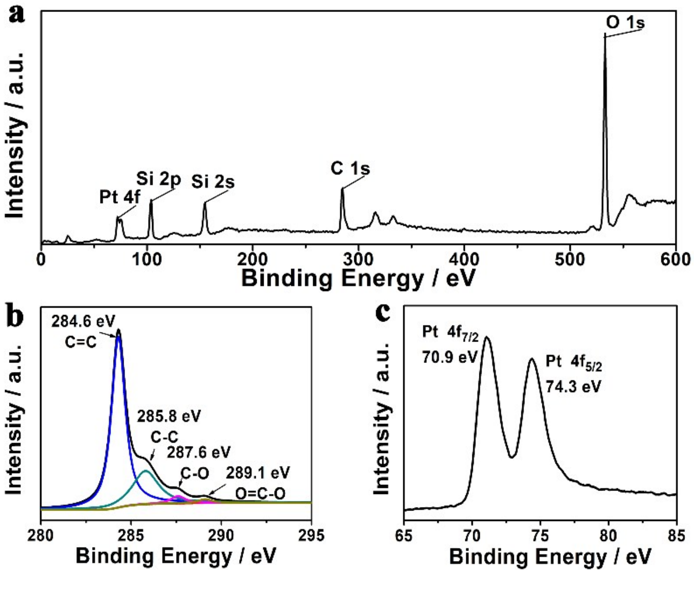

Fig. S2 XPS (a) survey spectra, (b) C 1s spectra and (c) Pt 4f spectra of PtNPs/eQG.

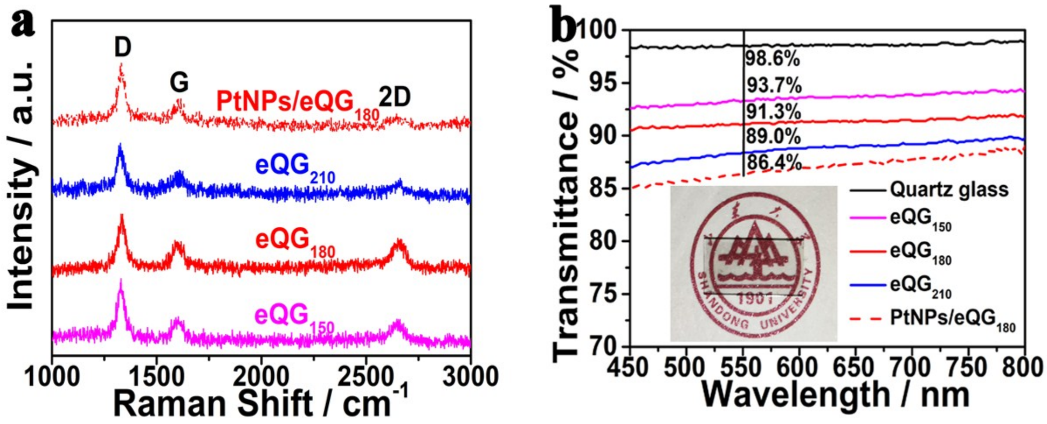

Fig. S3 (a) Raman spectra and (b) UV–vis spectra of eQG_{150}, eQG_{180}, eQG_{210} and PtNPs/eQG_{180}. Inset b is the photo of PtNPs loaded on eQG_{180} with good transmittance.

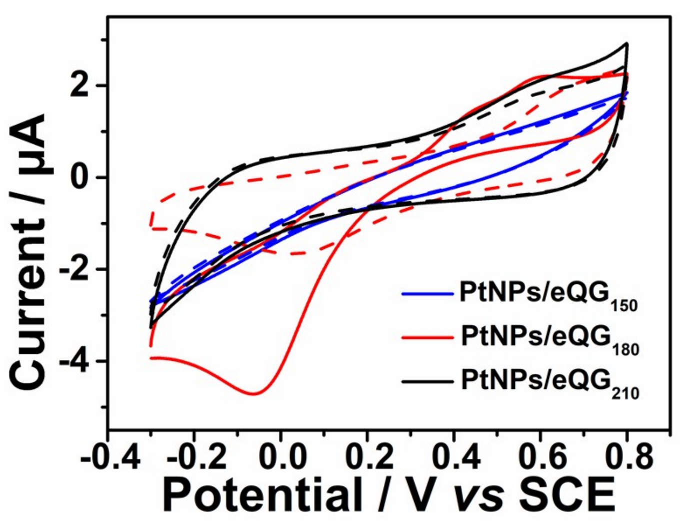

Fig. S4 Cyclic voltammogram curves of PtNPs/eQG_{150} (blue line), PtNPs/eQG_{180} (red line) and PtNPs/eQG_{210} (black line) in the presence (solid line) and the absence (dashed line) of 10 μM H_2O_2 with a scan rate of 50 mV/s.

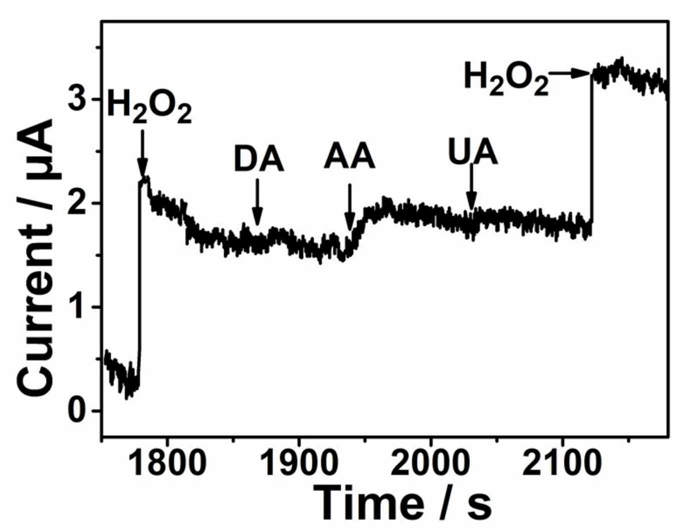

Fig. S5 Amperometric i-t curve of PtNPs/eQG upon intermittently adding H_2O_2 (0.05 mmol/L), dopamine (DA, 0.5 mmol/L), ascorbic acid (AA, 0.5 mmol/L) and uric acid (UA, 0.5 mmol/L) to phosphate buffer solution with a potential of 0.60 V.
